# Obstructive Sleep Apnea in Pregnancy: A Narrative Review

**DOI:** 10.7759/cureus.30387

**Published:** 2022-10-17

**Authors:** Surekha Tayade, Shikha Toshniwal

**Affiliations:** 1 Department of Obstetrics and Gynaecology, Jawaharlal Nehru Medical College, Datta Meghe Institute of Medical Sciences (Deemed to be University), Wardha, IND

**Keywords:** apnea–hypopnea index, continous positive airway pressure, pregnancy, sleep-disordered breathing, obstructive sleep apnea (osa)

## Abstract

Obstructive sleep apnea (OSA) is a sleep-related breathing disorder that remains underdiagnosed in pregnancy and can cause severe maternal and fetal complications. The prevalence of OSA in pregnancy had a wide variation and increases with increasing gestation. At-risk women are those with increased body mass index (BMI), increasing age, and chronic hypertension. Screening can be done by polysomnography but as it is expensive and necessitate overnight hospital stay, makes it even harder to diagnose and leads to delay in treatment. Once diagnosed, continuous positive airway pressure (CPAP) is the gold standard treatment. Early diagnosis and effective treatment can considerably improve the outcome. This review aims to discuss the diagnosis, treatment, and prognosis of OSA in pregnancy.

## Introduction and background

Obstructive sleep apnea (OSA) involves a reduction or cessation of airflow despite an effort to breathe. This condition is brought on by periodic collapses of the upper airway, which cause arousal, fragmented sleep, intermittent oxygen desaturation, and sympathetic activation [1]. This causes breathing to stop completely or partially for at least 10 seconds during sleep and might extend up to one minute. Effects of OSA include fluctuating oxygen levels, increased heart rate and blood pressure, increased risk of stroke, impaired glucose tolerance and insulin resistance, higher rate of heart disease-related death, impaired concentration, and mood swings [2] and all these can negatively impact pregnancy outcomes.

OSA In pregnancy

Pregnant women with OSA are most likely one of two different clinical phenotypes: either they already have OSA and become pregnant (chronic OSA), or they develop OSA during pregnancy (gestational OSA). Because of the physiological and hormonal changes that occur during pregnancy, as well as other comorbidities (multiple gestations, hypertensive disorders of pregnancy, or gestational diabetes) that may have occurred throughout pregnancy, women with gestational OSA may snore before becoming pregnant and experience a worsening airway obstruction. Pregnancy-related physiological changes that may put women at risk for OSA include upper airway edema, narrowing of the oropharyngeal diameter, increased Mallampati score [3], decreased functional residual capacity, increased oxygen consumption, and larger negative upper airway pressures brought on by elevated levels of the hormones estrogen and progesterone, which can cause or worsen sleep-disordered breathing (SDB) [4,5]. On the other hand, some pregnancy-related alterations, such as a predilection for the lateral sleep position and a higher respiratory rate brought on by hormonal changes may be beneficial. As the pregnancy goes on, these modifications change dynamically.

## Review

Methods

We searched PubMed database, MEDLINE, Web of Science, and Google Scholar using Medical Subject Headings (MeSH) terms obstructive sleep apnea, pregnancy, and sleep-disordered breathing, and found 424 articles (1976-2022) from which 27 relevant articles were included in the review. The articles included in the review are shown in Table 1.

**Table 1:** Relevant articles included in the review

AUTHOR	PLACE	YEAR
Somers et al. [1]	Iowa City, Iowa, United States	1995
Bourjeily et al. [4]	Providence*, *Rhode Island, United States	2011
Iczi et al. [5]	Edinburg, United Kingdom	2008
Pien et al. [6]	Philadelphia, United States	2014
Facco et al. [7]	Chicago, Illinois, United States	2017
Young et al. [8]	Wisconsin. United States	2002
Punjabi et al. [9]	Baltimore, Maryland, United States	2008
Jordan et al. [10]	Melbourne, Australia	2014
Pamidi et al. [11]	Canada	2016
Atilade et al. [12]	New York, United States	2013
Facco et al. [13]	Chicago, Illinois, United States	2012
Kapur et al. [14]	Washington, United States	2017
O’Brien et al. [15]	Michigan, United States	2012
Ghegan et al. [16]	South Carolina, United States	2006
Karakis et al. [17]	Maryland, United States	2012
Arnardottir et al. [18]	Philadelphia, United States	2009
Louis et al. [19]	Florida, United States	2014
Ge et al. [20]	China	2016
Spence et al. [21]	California, United States	2017
Pamidi et al. [22]	Canada	2018
Ding et al. [23]	China	2014
Louis et al. [24]	Florida, United States	2014
Pien et al. [25]	Philadelphia, United States	2005
Lamon and Habib [26]	North Carolina, United States	2016
Chung et al. [27]	Canada	2016
Zaremba et al. [28]	Boston, Massachusetts, United States	2015
Lalmand et al. [29]	Belgium	2017

Prevalence

There is little research on the prevalence of objectively diagnosed OSA in patients who are pregnant. Depending on gestational age and the method used to diagnose OSA, rates might range from 3-27% [6]. In one of the prospective studies that have been published, among the 3,132 nulliparous women who had objective testing for OSA, the prevalence of OSA was found to be 3.6% in the first trimester and rose during gestation, reaching rates as high as 26% at term. These observations are consistent with those of Pien et al. who, in a group of pregnant women who had overnight polysomnography at the two gestational periods, discovered an increase in the number of women with OSA from 10.5% in the first trimester to 26.7% in the third trimester [6,7].

Classification of OSA (American Academy of Sleep Medicine)

The classification of OSA is mentioned in Table 2 [2].

**Table 2:** Classification of obstructive sleep apnea OSA: obstructive sleep apnea

Classification of OSA	Apnea-Hypopnea Index	Involuntary sleepiness during activities requiring
Mild	5-15	Little attention
Moderate	15-30	Some attention
Severe	>30	More active attention

Risk factors

Men, older age, African-American race, obesity, craniofacial anomalies, pregnancy, and smoking are all known risk factors for OSA [8,9]. Type II diabetes, hypertension, cardiac arrhythmias, and cardiovascular disease are only a few of the co-morbid disorders that OSA is linked to [10]. The risk of OSA may be higher for women who had those risk factors before being pregnant. Additionally identified as risk factors in pregnancy by the current research are increasing gestation, increasing maternal age, increased BMI, chronic hypertension, and snoring (3x/week) [11-13].

Screening and diagnosis

Maternal symptoms like extreme daytime sleepiness or generalized fatigue, loud and frequent snoring, headaches, and/or observed maternal hypoxia in the absence of any cardiac or respiratory pathology should increase the suspicion of sleep apnea [14].

Diagnosis of OSA can be done through questionnaires like STOP (snoring, tiredness during daytime, observed apnea, and high blood pressure), STOP-BANG (body mass index (BMI), age, neck circumference, and gender), Epworth Sleepiness Scale, etc. In-lab overnight attended polysomnography (PSG) is the gold standard for the diagnosis of OSA [15]. However, many patients may find it impractical as in-lab PSG is expensive and necessitates an overnight hospital stay.

Because of these difficulties, a lot of sleep specialists are turning to portable sleep testing done at home as a workable solution for some populations. Home sleep tests, on the other hand, are unsupervised and don't use EEG to assess sleep duration; thus, they are more likely to understate the severity of sleep apnea or give falsely negative results [15-17]. To estimate actual sleep time, routine EEG provides direct clinical observation in addition to electrophysiologic and cardiorespiratory monitoring; this feature is absent from most home testing [18].

Maternal complications

OSA is associated with endothelial dysfunction, which has been implicated in the development of gestational diabetes and gestational hypertension [7,11,19].

SDB in early and mid-pregnancy were associated with preeclampsia (adjusted odds ratio (aOR) 1.94 (95%CI 1.07-3.51) and 1.95 (95%CI 1.18-3.23)), and gestational diabetes (GDM) (aOR 3.47 (95%CI 1.95-6.19) and 2.79 (95%CI 1.63-4.77)) [7]. The findings of that study were similar to the findings of other smaller retrospective and prospective cohort studies that found a two-fold increased adjusted odds of preeclampsia and a nearly two-fold increased adjusted odds of gestational diabetes in association with SDB or OSA, in two meta-analyses of the existing studies [11,20].

A study by Spence et al. concluded that women with an OSA diagnosis were more likely to have a cesarean delivery (aOR, 1.60; 95%CI, 1.06-2.40), gestational hypertension, (aOR, 2.46; 95%CI, 1.30-4.68), preeclampsia (aOR, 2.42; 95%CI, 1.43-4.09), and preterm delivery (aOR, 1.90; 95%CI, 1.09-3.30) [21].

Louis et al. conducted a large, national inpatient database study, which showed that pregnant women with a diagnosis of OSA during their hospital admission at delivery were at significantly increased risk of having cardiomyopathy (aOR = 9.0 (95%CI, 7.47-10.87)), congestive heart failure (aOR = 8.94 (95%CI, 7.45-10.73)), and pulmonary embolism (aOR = 4.5 (95%CI, 2.3-8.9)). This study also showed a five-fold increase in in-hospital mortality during pregnancy or delivery in women with OSA [9].

Neonatal complications

Low birth weight and small-for-gestational-age newborns are 1.5-2 times more common in mothers who have OSA. These results hold up even after taking into account maternal comorbidities like hypertension that increase the risk of growth restriction [22,23]. No research has found an association between sleep apnea and an increased risk of fetal death or miscarriage [20].

Treatment

Antepartum Care

When treating pregnant women with OSA, a multidisciplinary strategy involving a sleep medicine specialist and an anesthetic should be used and should last until the postpartum period [24]. The standard management option for moderate to severe OSA cases and a suitable alternative for mild sleep apnea is CPAP. Patients who use CPAP receive a consistent flow of compressed air through a mask they wear while they sleep. This airflow keeps the airway open, reducing breathing pauses and bringing oxygen levels back to normal.

To optimize CPAP settings, women with known OSA who become pregnant should be examined by a sleep medicine specialist. Through regular CPAP usage, it is hoped to attain normalized apnea-hypopnea index (AHI) and oxygenation throughout gestation. As CPAP requirements may rise with increasing gestation, follow-up appointments or CPAP equipment with an automatic titrating feature can be helpful. Patients with moderate to severe OSA may also have pulmonary hypertension or co-morbid cardiovascular illness; hence, echocardiography should be considered as well. Obstetric clinicians should concentrate on the early detection or prevention of these problems and be aware of the risk of diabetes and hypertensive disorders. Additionally, switching from back to side sleeping may be beneficial for people with mild OSA [6,7,25].

Women who may have OSA but have not yet received a diagnosis should be referred to a sleep medicine specialist for assessment.

Intrapartum and Postpartum Management

Additionally, women with OSA are more likely to have co-morbid illnesses that increase their risk for cesarean births [12]. According to the recommendations of the American Society of Anesthesiologists, preoperative OSA assessment and therapy is ideal for surgical patients. Having sleep apnea increases the likelihood of difficult intubation and breathing during surgery for pregnant women. The gold-standard anesthetic for cesarean deliveries, neuraxial anesthesia, might be technically challenging when OSA is coexisting with severe obesity. This might make switching to general anesthesia more necessary [26]. An evaluation of the airway and a neuraxial anesthetic placement should take place during the pre-operative anesthesia consultation. These women should undergo ongoing pulse oximetry monitoring after leaving the recovery room because they run the risk of experiencing postoperative respiratory suppression [24,27].

As this was linked to a reduction in the number of apnea and hypopnea events in a study of post-partum women, patients should be advised to maintain a 45-degree head elevation and avoid a supine position [28]. Anti-emetics, antihistamines, anxiolytics, and sleep aids are sedative drugs that should be avoided or used very rarely under controlled settings, especially when used with opioids. Patient-controlled systemic opioids should be used extremely cautiously, whereas basal dosing and standing orders for narcotics should be avoided [29].

## Conclusions

OSA in pregnancy is an under-recognized disorder which is frequently missed and can cause fetal and maternal complications. These women are at a higher risk of complications like gestational hypertension, gestational diabetes mellitus, increased incidence of cesarean section, preterm delivery, etc. and complications due to anesthesia. A lack of awareness and efficient screening tools makes diagnosis and treatment challenging. Increased awareness with early diagnosis, treatment, and perioperative management could improve maternal and fetal outcomes in these pregnancies. In India, as pregnancy is often the time many young rural women seek hospital care, this is the best time for early detection of OSA in pregnancy.
